# Danger of Food and Drink

**Published:** 1886-01

**Authors:** 


### DANGER IN FOOD AND DRINK.

The *Century* has, for the month of December, a most excellent article on the faith cure. It also has a very important article on "Dangers in Food and Drink," in which the author gives some of the facts discovered by the investigations of the New York City Health Department. It shows a fearful state of things for coffee drinkers. He says: "Polishing was originally practiced without the addition of any mineral substance to improve the general appearance of raw coffee; but it was accidentally discovered that the addition of small amounts of pulverized soapstone effects a much more decided improvement. This led to the use of mineral substances and pigments to affect the color, until now coffee can be 'painted' any desired shade by those skilled in this brand, just as one can get from a dyer any desired shade on fabrics. A list of the substances used in this 'painting' may here find a place: gum arabic, venetian red, French chalk or soapstone, silesia blue, crome yellow, Prussian blue, turmeric, burnt umber, yellow ochre, drop black.

The silesia blue consists of a mixture of Prussian blue and baryte. In the sample examined, a small amount of lead (probably there a white lead) was detected. The "drop-black is ground bone black. The other names require no explanation."

We have laws against this drugging of coffee, but it is still practiced to a very great extent. There is, probably, ten pounds of mixed and poisoned offee sold to one of the pure article. It is so common that we never hear of a case of prosecution for mixing or selling the poisonous compounds. The Germans are very fond of what is known, in their language, as "Eier Nudeln," a yellow vermicilli, the color being supposed to be from the mixture of eggs with the the flour, mixed in the manufacture, and which was, in the first place, eggs, but since eggs have become more valuable they have substituted Turmein Marten's yellow (a coal tar dye) and chrome yellow (chromate of lead). The last is the more dangerous, and one case of lead poisoning has been traced directly to that cause.

The writer in the *Century* gives the poisons in mustard; copper in giving a bright green tint to pickles and other preserved green vegetables. Here is a paragraph that is particularly entertaining:

"The westerner who visits this city on business or pleasure may 'forget to taste' the Croton water; but how would he feel if he were told that the sparkling effervescent water which he drank with his claret at some fashionable club, even though it be some well-known foreign brand with label and cork-fastening looking as though the bottle had just come from abroad, was water drawn from a well sunk on Manhattan Island, and was contaminated with the drainage of some of its busy streets and leaky sewers. Yet that has been often the case.

